# *Metschnikowia* Species Share a Pool of Diverse rRNA Genes Differing in Regions That Determine Hairpin-Loop Structures and Evolve by Reticulation

**DOI:** 10.1371/journal.pone.0067384

**Published:** 2013-06-21

**Authors:** Matthias Sipiczki, Walter P. Pfliegler, Imre J. Holb

**Affiliations:** 1 Department of Genetics and Applied Microbiology, University of Debrecen, Debrecen, Hungary; 2 Plant Protection Institute, Hungarian Academy of Sciences, and University of Debrecen, Debrecen, Hungary; University of Ottawa, Canada

## Abstract

Modern taxonomy of yeasts is mainly based on phylogenetic analysis of conserved DNA and protein sequences. By far the most frequently used sequences are those of the repeats of the chromosomal rDNA array. It is generally accepted that the rDNA repeats of a genome have identical sequences due to the phenomenon of sequence homogenisation and can thus be used for identification and barcoding of species. Here we show that the rDNA arrays of the type strains of *Metschnikowia andauensis* and *M. fructicola* are not homogenised. Both have arrays consisting of diverse repeats that differ from each other in the D1/D2 domains by up to 18 and 25 substitutions. The variable sites are concentrated in two regions that correspond to back-folding stretches of hairpin loops in the predicted secondary structure of the RNA molecules. The substitutions do not alter significantly the overall hairpin-loop structure due to wobble base pairing at sites of C-T transitions and compensatory mutations in the complementary strand of the hairpin stem. The phylogenetic and network analyses of the cloned sequences revealed that the repeats had not evolved in a vertical tree-like way but reticulation might have shaped the rDNA arrays of both strains. The neighbour-net analysis of all cloned sequences of the type strains and the database sequences of different strains further showed that these species share a continuous pool of diverse repeats that appear to evolve by reticulate evolution.

## Introduction

The wide-spread application of DNA sequence analysis to taxonomy and phylogenetic studies have shown that phenotypic traits are poor indicators of genetic and evolutionary relatedness among yeast species and higher taxonomic groups. Therefore contemporary yeast taxonomy is mainly based on the comparative analysis of conserved parts of the genomes such as the nuclear rRNA operon, genes coding for components of the transcriptionary and translationary machineries, their combinations (e.g. [1] and references therein) and genes encoding cytoskeletal components [2], [3]. In phylogenetic analysis of higher taxonomic units, a multigenic approach is preferred (e.g. [1]). Conserved domains of transcription factors also seem to be suitable for the assessment of phylogenetic relationships [4]. By far the most frequently used sequences are those of the domains 1 and 2 (D1/D2) of the large subunit (LSU, 26S) rDNA and the ITS1-5.8S-ITS2 regions of the rDNA repeats. Recently an international consortium proposed the ITS sequence for barcoding of fungi [5]. Time will show whether it can also be adopted as the major barcoding sequence for ascomycetous yeasts. However, for the time being, most yeast biologists consider it too variable and prefer the more conserved large subunit rRNA gene for species delimitation (for a review see [6]). In phylogenetic analyses of these sequences the large and rapidly expanding group of *Metschnikowia* and related anamorphs usually formed a monophyletic but heterogeneous clade of Saccharomycotina (e.g. [7]). Within the clade a group of species related to the pulcherrimin-producing *M. pulcherrima* form a well-separated, compact subclade with high statistical support [7], [8], [9].

The phylogenetic trees of the *Metschnikowia* clade inferred from D1/D2 domain sequences of the type strains of the species included *M. andauensis* and *M. fructicola* which, however, contain ambiguous nucleotides in their database sequences [3], [10]. As the methods used for estimation of phylogenetic relations are based on differences between nucleotide sequences, ambiguous positions can lead to incorrect conclusions and preclude the correct taxonomic assignment of new isolates. In spite of this, the *M. andauensis* and *M. fructicola* sequences were also used for the demonstration of phylogenetic separation and delineation of new *Metschnikowia* species (e.g. [8]). In a project aimed at the isolation of novel pulcherrimin-producing *Metschnikowia* strains suitable for bioprotection (e.g. [11]), we frequently encountered the problem that most isolates could not be assigned to any known species on the basis of their D1/D2 sequences although they were fairly similar to one or the other species of the subclade. They did not show sequence identity to any of the type strains of known species and did not form a compact group either, indicating that they did not represent novel, distinct species. In principle, ambiguous nucleotides in a sequence can be attributed to sequencing errors or to heterogeneity in the amplified DNA caused by the presence of two or more fragments of different sequences. Both reasons could account for the ambiguity of the database sequences of the type strains of *M. andauensis* and *M. fructicola* but the fact that their ambiguous nucleotides are not scattered randomly makes the latter possibility more likely.

In our quest to elucidate the reason of ambiguous nucleotides, we resequenced the D1/D2 domains of both strains but before sequencing we cloned fragments from the amplified DNA. In this paper we report on the results of the analysis that revealed an unexpectedly high level of heterogeneity and polymorphism in sequence and secondary structure of the LSU rRNA domains. We will use “heterogeneity” to designate the presence of different versions of paralogs (and their nucleotides) and “polymorphism” to refer to the presence of versions (alleles) of the same ortholog in different strains. When phylogenetic trees were constructed, the two sets of cloned sequences formed intermixed branches, indicating that reticulate evolution may have occurred in the history of the strains. In situations where reticulate processes can be suspected, bifurcating trees based on a model of evolution dominated by mutations and speciation events can be an inappropriate representation of the phylogenetic history. In such cases more general graphs such as phylogenetic networks can be more useful as they allow the visualization of horizontal events and competing evolutionary scenarios within a single structure (for reviews see [12], [13]). Therefore we reanalysed the sequences using the splits-based neighbour-net approach [14] and concluded that intrastrain, interstrain and interspecies reticulation events must have shaped their rDNA arrays.

## Materials and Methods

### Strains and Culture Conditions

The yeast type strains *M. andauensis* CBS 10809^T^ and *M. fructicola* CBS 8853^T^ were obtained from the CBS (Centraalbureau voor Schimmelcultures, Utrecht, the Netherlands) collection. The strains were routinely maintained on YPGA plates. To obtain single-cell clones, cells of their overnight cultures grown in the liquid medium YPGL were plated out onto YPGA plates and incubated at 30°C for 4 days. All strains and yeast clones are listed in Table 1. The composition of the media is described in [15].

**Table 1:** List of strains and sequences.

Strain	Single-cell clone	Cloned sequence	Accession number	Source
*M. andauensis* CBS 10809^T^ (11–1120)			AJ745110	[3]
		a77	KC411953	This study
		a78	KC411954	This study
	a	aa20	KC411955	This study
		aa23	KC411956	This study
		aa23a	KC411957	This study
	b	ab24	KC411958	This study
		ab27	KC411959	This study
*M. andauensis* HA 1622			AJ745108	[3]
*M. fructicola* CBS 8853^T^ (11–579)			AF360542	[10]
	b	fb1	KC411962	This study
		fb3	KC411963	This study
		fb6	KC411964	This study
		fb9	KC411965	This study
		fb10	KC411966	This study
		fb11	KC411967	This study
	c	fc15	KC411968	This study
		fc17	KC411969	This study
		fc21	KC411970	This study
	39a	f39a1	KC411960	This study
	39b	f39b2	KC411961	This study
*Candida picachoensis* CBS 9804^T^			AY452039	[59]
*Escherichia coli*			J01695	[60]

T type strain.

### Amplification, Cloning and Sequencing of rDNA

Nuclear DNA was isolated from overnight cultures grown in YPGL broth as described previously [16]. The isolated DNA was used for the amplification of the D1/D2 domains of the large subunit rRNA genes with the primers NL-1 and NL-4 [17]. The PCR products were used for random cloning of D1/D2 fragments into the pGEM-T Easy Vector, following the manufacturer’s instructions (Promega, Madison, WI). Bacterial colonies were randomly selected from the transformants. The plasmids were extracted from the bacterial clones and checked for the size of the inserts by reamplification with the primer pair NL-1 and NL-4. The inserts were sequenced in both directions using the same primers. The 499 nt-long sequences covering the chromosomal regions between the amplification primers were deposited in GenBank under accession numbers listed in Table 1.

### Sequence and Secondary Structure Analysis

The cloned sequences were compared with each other using the bl2seq algorithm available in NCBI (http://blast.ncbi.nlm.nih.gov/Blast.cgi). Each sequence was tested for similarity/identity to sequences deposited in databases by Megablast-search on the NCBI web site. For aligning of multiple sequences, the Clustal W 1.7 [18] algorithm was used. Since certain sequences extracted from databases were longer than the cloned sequences, they had overhangs not aligning with the cloned sequences. These regions were removed after the first alignment and from the trimmed sequences new alignments were produced for further analysis. These multiple alignments were used for the identification of sites with variable nucleotides. The sites were numbered arbitrarily starting with the first nucleotide located behind the end of primer NL1. WebLogos [19] for the variable segments were generated from the multiple alignments with the tool available at http://weblogo.berkeley.edu/logo.cgi.

Models of rRNA secondary structures were predicted from nucleotide sequences with the programme RNAstructure version 5.4 [20], which folds RNA based on the principle of minimizing free energy [21]. First the entire D1/D2 sequences were analysed to identify the structures formed by the variable regions. Both were parts of hairpin stems. Then all segments not involved in the stems were removed from the nucleotide sequences and new secondary structures were generated. The variable sites were localised in the structures and the potential effect of the substitutions on the secondary structure was examined by comparing the structures generated from the individual clones.

### Phylogenetic Analysis

For phylogenetic analysis neighbour-joining, maximum-parsimony, maximum-likelihood and Bayesian methods were used. The neighbour-joining and maximum-parsimony trees were constructed with the Phylip version 3.67 software package [22]. In the neighbour-joining analysis, the F84 model of nucleotide substitutions [23] was used for computing distance matrices. Confidence limits were estimated from bootstrap analysis based on 1000 replications using Seqboot and Consence (majority-rule) programmes of the package. The maximum-likelihood tree was generated with the PhyML 3.0 algorithm [24] in combination with the Seqboot and Consense tools from the Phylip package. In this analysis settings were made according to the best model suggested by the Akaike Information Criterion (AIC) in jModelTest version 2.0.2. [25]. Bayesian inference of phylogeny was done using MrBayes 3.2. [26] with the General-Time-Reversible (GTR) substitution model for nucleotide sequences [27] and gamma-shaped rate variation with a proportion of invariable sites. The number of discrete categories used to approximate the gamma distribution was set to 4. The MCMC processes were set so that four chains (one cold and three heated; setting a default temperature for heating the chains) were run simultaneously for 1,000,000 generations. The average standard deviation of split frequencies was P = 0.004653, indicating that a convergence had occurred (P-value of <0.05). Trees were sampled every 100 generations. The first 25% of samples were discarded from the cold chain as burn-in. Bayesian posterior probability of the branches was estimated from 12899 trees. The trees were visualized with the TreeView [28] and FigTree (http://tree.bio.ed.ac.uk/) programmes.

### Network Analysis

Network analysis was performed using the SplitsTree4 V4.12.8 package [14], taking as input the Clustal alignments of the D1/D2 domains of the rDNA repeat units. For distance calculation the distance estimation method K3ST (Kimura’s three-substitution-types; [29]) was used. To construct cluster networks in form of rooted rectangular phylogrammes, the sequence AY452039 of *Candida picachoensis* CBS 9804^T^ was used as outgroup. This species is close enough to the *M. pulcherrima* group [7] to have a D1/D2 sequence moderately related to the analysed sequences but far enough to be an uncontroversial outgroup. To generate neighbor-nets, Equal Angle setting was chosen and the sequence used as outgroup in cluster networks was excluded from the analysis, as its inclusion had little effect on the overall structure (the neighbor nets are unrooted networks) of the network and the layout of the network was improved by its exclusion. To test the aligned sequences for recombination we used the Phi test of Bruen et al. [30] as available in the SplitsTree4 package.

## Results

### rDNA Heterogeneity and Polymorphism

From genomic DNA of the *M. andauensis* and *M. fructicola* type strains, we cloned 7 and 11 full-lengths PCR-amplified D1/D2 fragments. Two *M. andauensis* fragments were cloned directly from the culture purchased from CBS, and five clones were obtained from two single-cell yeast clones of the species. The *M. fructicola* D1/D2 clones were obtained from four single-cell yeast clones. The cloned DNA fragments were sequenced from both ends and the sequences were deposited in the GenBank database (Table 1).

Pairwise Blast comparison revealed that the majority of the cloned fragments had unique sequences. There were only two pairs with identical sequences: one pair (aa20 and ab24) in the *M. andauensis* set and one pair (f39b and fb6) in the *M. fructicola* set. The number of substitutions ranged from 1 to 18 in *M. andauensis* and 2 to 25 in *M. fructicola*. The largest differences corresponding to 3.6% and 5.0%, respectively, exceeded 1%, the value generally considered as the limit of variability among conspecific strains of most ascomycetous yeasts [31]. When the clones of the two strains were compared, no identical sequences were identified and differences were detected at 2 to 25 sites. Thus, the range of variability was practically identical at the intrastrain and interstrain levels. Interestingly, there were clones in both sets for which the most similar partner was found among the clones of the other strain (e.g. aa13a – fb9, aa20– fc21, a78– fc15).

**Figure 1:**
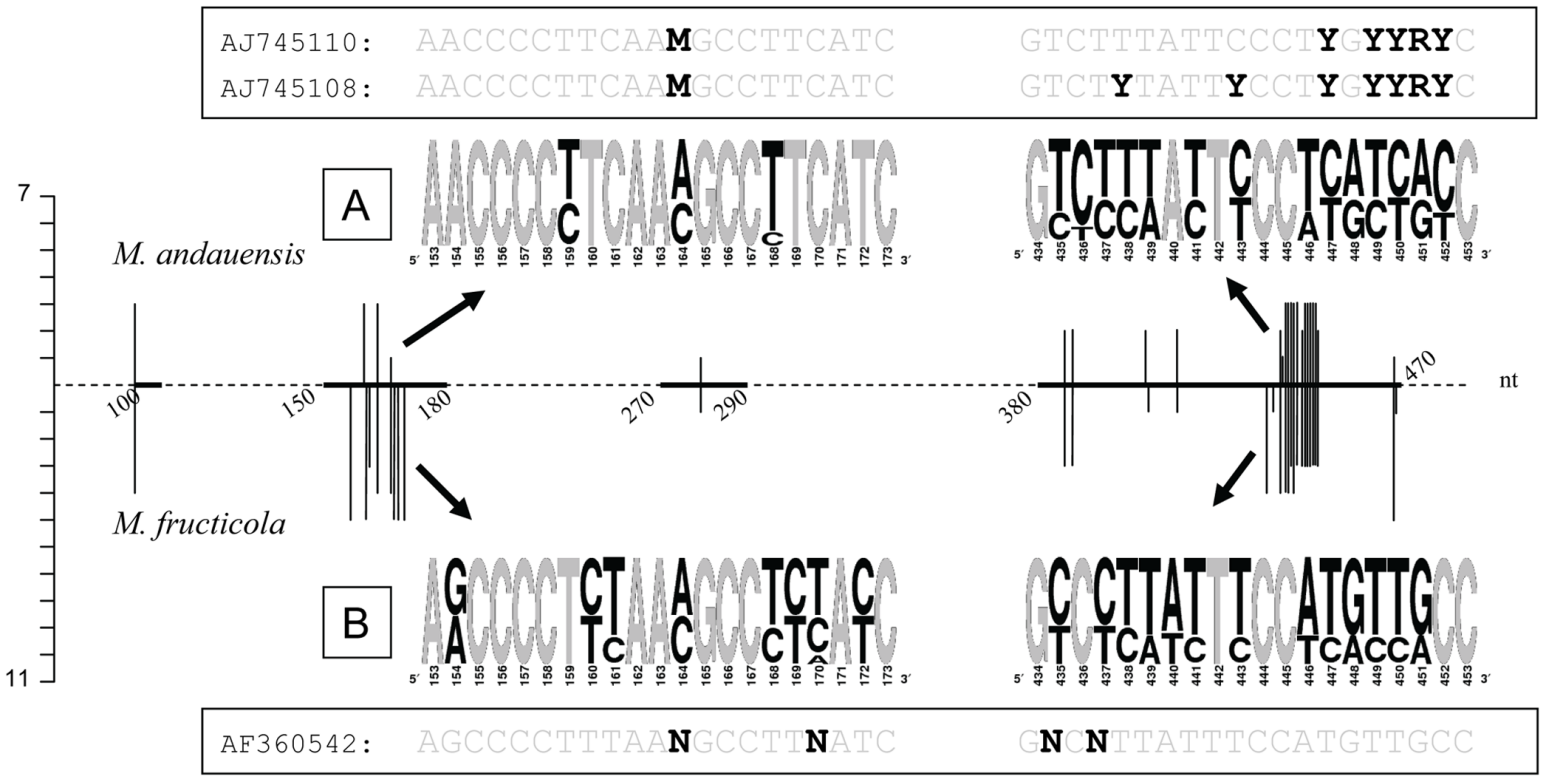
Variable sites in the D1/D2 domains of the type strains of *M. andauensis* and *M. fructicola*. The graph in the middle of the figure shows the location of the variable sites. The horizontal line represents the D1/D2 domain. For better orientation, the distance (in number of nucleotides) of certain sites from the 3′ end of the NL1 primer is shown. Each variable site is marked with a vertical line above (*M. andauensis*) or below (*M. fructicola*) the D1/D2 line. The height of a vertical line shows the number of cloned alleles which had a nucleotide at that position different from the nucleotide of the majority of the *M. andauensis* or the *M. fructicola* clones. The sequences of the regions with high density of variable sites are shown in Weblogos (A and B). In the Weblogos, the nucleotides of the variable sites are highlighted with black colour. The framed sequences are the corresponding segments of Genbank sequences of the type strains of *M. andauensis* and *M. fructicola*. The *M. andauensis* frame also contains the other sequence of the species available in the database. In the framed sequences the symbols of ambiguous nucleotides are highlighted with black colour.

The multiple alignment of all cloned sequences identified variability (mostly dimorphism) at 35 sites (Tables 2 and 3). The variable positions were not distributed evenly along the entire length of the D1/D2 domains (Fig. 1). The majority of them grouped in two short regions (Fig. 1) which we will call variable region 1 (VR1) and variable region 2 (VR2) throughout this paper. Nine variable sites of the D1 domain formed VR1 and 16 sites of the D2 domain comprised VR2. In these sites usually two nucleotides alternated; there were only two sites where more than two different nucleotides occurred when all cloned sequences were compared. The histogram and the Weblogos in Fig. 1 show the proportions of the alternating nucleotides. The nucleotides of the two sites (159 and 432) which differed only in single clones might be attributed to sequencing errors. The majority of variable sites varied in both strains (Tables 2 and 3 and Weblogos in Fig. 1). Transitions were much more abundant than transversions: 79% in *M. andauensis* and 82% in *M. fructicola*. Among transitions the T-C substitutions predominated (74% and 65%, respectively).

**Table 2:** Nucleotides at variable positions in the D1 domain.

Strain/Clone	Nucleotides at variable positions in D1										
	103	154	159	160	161	164	168	169	170	172	280
*M. andauensis*											
Clones	A/G	A	T/C	T	C	A/C	T/C	T	C	T	A/C
Type^1^	**R**	A	T	T	C	**M**	T	T	C	T	A
Reference^2^	**R**	A	T	T	C	**M**	T	T	C	T	A
*M. fructicola*											
Clones	A/G	A/G	T	T/C	T/C	A/C	T/C	T/C	T/C/A	T/C	A/G
Type^3^	**N**	G	T	T	T	**N**	T	T	**N**	T	A

IUPAC degenerate base symbols: N, any base; M, amino (A or C); R, purine (A or G). ^1^ *M*. *andauensis* CBS 10809^T^ sequence: AJ745110.

2 *M*. *andauensis* HA 1622 sequence: AJ745108.

3 *M*. *fructicola* CBS 8853^T^ sequence: AF360542.

**Table 3:** Nucleotides at variable positions in the D2 domain.

Strain/Clone	Nucleotides at variable positions in D2																							
	389	391	401	402	415	428	432	435	436	437	438	439	440	441	443	446	447	448	449	450	451	452	466	467
*M. andauensis*																								
Clones	A/G	A/G	T/C	G	T/C	A	C	T/C	T/C	T/C	T/C	A/T	A	T/C	T/C	A/T	T/C	A/G	T/C	T/C	A/G	T/C	A/T	T
Type^1^	G	G	C	G	T	A	C	T	C	T	T	T	A	T	C	T	**Y**	G	**Y**	**Y**	**R**	**Y**	A	T
Reference^2^	**R**	**R**	C	G	T	A	C	T	C	T	**Y**	T	A	T	**Y**	T	**Y**	G	**Y**	**Y**	**R**	**Y**	A	T
*M. fructicola*																								
Clones	A/G	A/G	C	A/G	T/C	A/C/T	T/C	T/C	C	T/C	T/C	A/T	A/T/−	T/C	T/C	A/T	T/C	A/G	T/C	T/C	A/G	C	A/T	T/−
Type^3^	A	A	C	G	T	**N**	C	**N**	C	**N**	T	T	A	T	T	A	T	G	T	T	G	C	T	T

IUPAC degenerate base symbols: N, any base; R, purine (A or G); Y, pyrimidine (C or T).

1 *M*. *andauensis* CBS 10809^T^ sequence: AJ745110.

2 *M*. *andauensis* HA 1622 sequence: AJ745108.

3 *M*. *fructicola* CBS 8853^T^ sequence: AF360542.

Comparison of the cloned sequences with the database D1/D2 sequences of the type strains (AJ745110 and AF360542) confirmed our hypothesis that the ambiguous nucleotides of the type strains could be due to heterogeneity of the PCR products used by the depositors for sequencing. It is evident from Fig. 1 and Tables 2 and 3 that all their ambiguous nucleotides corresponded to sites variable in the clones and their ambiguity symbols (M, R, Y and N) were in agreement with the alternating nucleotides (A/C, A/G, C/T and any base, respectively).

The Megablast search in GenBank with the cloned sequences did not identify identical sequences with the exception of fc21 of *M. fructicola*. fc21 showed 100% identity with EU386763 deposited as the D1/D2 domain of the taxonomically uncharacterized strain *Metschnikowia* aff. *fructicola* C723 isolated from wine in China. This entry was shorter by 24 nucleotides than the query sequence but its missing 3′ end overlapped with the non-variable terminal region of the D2 domain. The other clones differed by 1–7 substitutions from the most similar database entries deposited under various, mostly uncertain taxonomic names such as *Metchnikowia* aff. *fructicola* and *Metschnikowia* sp. A search by name in the database resulted in the identification of the sequences of the type strains and additional 1 *M. andauensis* and 7 *M. fructicola* strains (Table 1). The *M. andauensis* sequence was the one used instead of the type-strain sequence in the phylogenetic analysis of the *Metschnikowia* clade by Lachance [7] and had more ambiguous nucleotides than the type-strain sequence. The *M. fructicola* sequences had no ambiguous sites but differed by various numbers of substitutions from all cloned sequences (see neighbor-net analysis below).

### Secondary Structure Analysis

**Figure 2:**
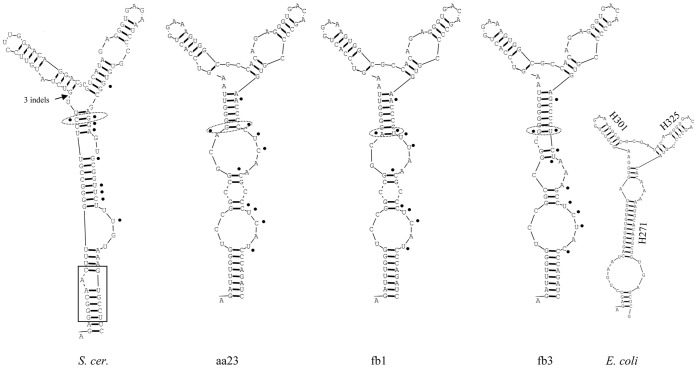
Predicted secondary structures of D1 hairpin-stem loops. Only examples of the *M. andauensis* (aa23) and *M. fructicola* (fb1 and fb3) clones are shown. The variable sites and their equivalents in the *S. cerevisiae* molecule (*S. cer.*) are marked with dots. The boxed region is the helical segment missing in the cloned *Metschnikowia* sequences. For *E. coli* helix nomenclature see http://www.rna.icmb.utexas.edu/.

**Figure 3:**
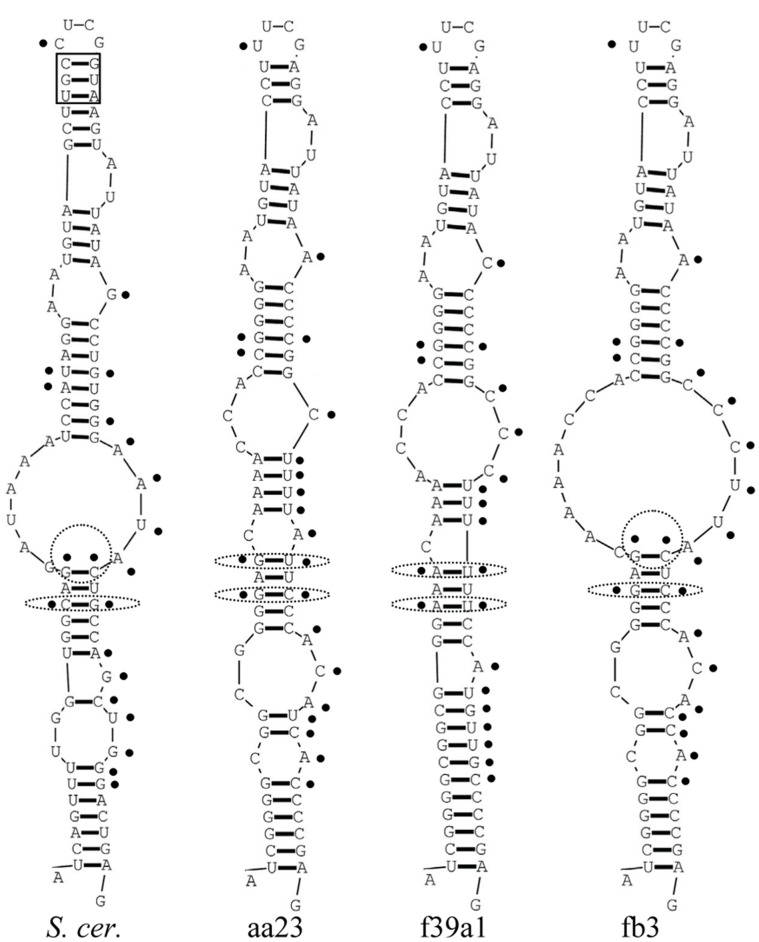
Predicted secondary structures of D2 hairpin-stem loops. Only examples of the *M. andauensis* (aa23) and the *M. fructicola* (f39a1 and fb3) clones are shown. Dots mark the variable sites and their equivalents in the *S. cerevisiae* molecule (*S. cer.*). The helical segment of the *S. cerevisiae* hairpin missing in *Metschnikowia* is boxed.

The question arises as to whether the substitutions in the VR1 and VR2 regions can affect the structure of the mature RNA molecules. To examine whether the sequence differences of the cloned D1/D2 domains entailed alterations in the structure of the encoded RNA molecules, we generated secondary structures for all cloned D1/D2 domains and compared them with those of the corresponding parts of the *Saccharomyces cerevisiae* large subunit (26S) rRNA and the *Escherichia coli* large subunit (23S) rRNA molecules. The cloned sequences showed similar structures, in which both variable regions were involved in helical stems of hairpin loops (stem-loops) (Figs. 2 and 3). The corresponding NL1-NL4-flanked region of the *S. cerevisiae* rRNA sequence produced similar structures but its stem-loops were slightly longer due to two short helical stretches (boxed in Figs. 2 and 3) missing in all *Metschnikowia* clones. The *S. cerevisiae* and *E. coli* stems (Figs. 2 and 3) produced here were essentially identical to the corresponding stems in the secondary models of the complete rRNA sequences available in http://www.rna.icmb.utexas.edu/.

The VR1 regions of all cloned sequences formed similar stem-loops that did not differ significantly from the corresponding hairpins of *S. cerevisiae* and *E. coli* either (examples are shown in Fig. 2). Structural variability was observed only in the stem parts, apparently due to the variable sites that grouped in the back-folding strands of the helices. The only exception was site 103 which was in the complementary strand. Similar topological variability and grouping of variable sites in the back-folding strands were also observed in the hairpin loops that included the VR2 segments (examples are shown in Fig. 3). These were formed from the equivalent of the large expansion segment of the *S. cerevisiae* molecule which has no counterpart in *E. coli* [32], so we could not generate a corresponding *E. coli* hairpin loop. These *Metschnikowia* hairpins had 4 variable sites (389, 391, 401 and 402) in the complementing helix and one variable position (415) was in the loop. Interestingly, the loop sequences of both types of *Metschnikowia* hairpins were more similar to those of *E. coli* than to those of *S. cerevisiae.* It has to be mentioned here that a previous study detected variable sites in the counterpart of this loop in *Clavispora* strains [33], a genus related to *Metschnikowia*.

### Non-canonical Base Pairing and Compensatory Base-pair Changes

As described above, both highly variable regions were located in the back-folding 3′ strands of the hairpins and almost all nucleotides of the variable sites paired with nucleotides of stable positions. This implies that each substitution at a variable site could alter the helical structure of its stem because it can disrupt the normal base pairing. Consistent with this, the hairpins of the clones showed variable patterns of paired and unpaired stretches (Figs. 2 and 3). However, not all nucleotide substitutions caused changes in the secondary structures (note that the figures show only examples). The structural neutrality of certain nucleotide changes could be attributed to non-canonical base pairing referred to as wobble base paring. Due to this peculiarity of RNA, guanine can pair not only with cytosine but also with uracil in the RNA helix [34]. Thus, the substitution of cytosine by thymine in the DNA sequence does not necessarily affect the structure of the RNA helix. Many variable sites of VR1 and VR2 (e.g. sites 160, 161, 169, 435, 437, 441, 448, 449 and 452) paired with stable guanines in the hybridising segment of the hairpin stems. Most of them had either T(U) or C in the cloned sequences. Their transitions did not alter the stem structure, confirming that wobble pairing of nucleotides did neutralise many substitutions indeed. So the structural variability could be ascribed to substitutions in sites, where wobble pairing was not possible. However, even in their case, not all nucleotide changes had structural effects. We noticed 3 variable sites in the back-folding stretches of the hairpins that paired with variable sites in the complementary sequences of the stems: 103–159, 389–443 and 391–441. As shown in Tables 4 and 5, in the majority of the cloned sequences these sites remained paired in spite of the nucleotide changes. Apparently, these site pairs must have mutated in a coordinated way to preserve their ability to pair.

**Table 4:** Compensatory nucleotide substitutions in VR1.

Type	Nucleotides at positions		Clones	Database sequences
	103	159		
I	A	T	a78, aa23a, ab27, fc15, fc17, fb1, fb9	JQ771743, EU441891, EU441900,
II	G	T	a77, f39a1, f39b2, fb3, fb6, fb10, fb11, fc21	HM191666, EU4411890, GQ281759, HQ658858
III	A	C	aa23	–
IV	G	C	aa20, ab24	–
V	R or N	T	–	AJ745110^T^, AF360542^T^

T type strain.

**Table 5:** Compensatory nucleotide substitutions in VR2.

Type	Nucleotides at positions		Clones	Database sequences
	389–391	443–441		
I	AAA	TTT	a78, aa20, ab24, fb1, fb6, fb10, fc21, fc17, fc15,f39a1, f39b2	AF360542^T^, JQ771743, EU441891, HM191666, EU441900
II	AAA	CTT	–	EU4411890, HQ658858
III	AAA	TTC	–	GQ281759
IV	AAA	CTC	a77, ab27	–
V	GAG	CTC	aa23a, fb3, fb11, fb9	–
VI	GAG	CTT	aa23	AJ745110^T^

T type strain.

### Phylogenetic Analysis of Heterogenic and Polymorphic rDNA Units

**Figure 4:**
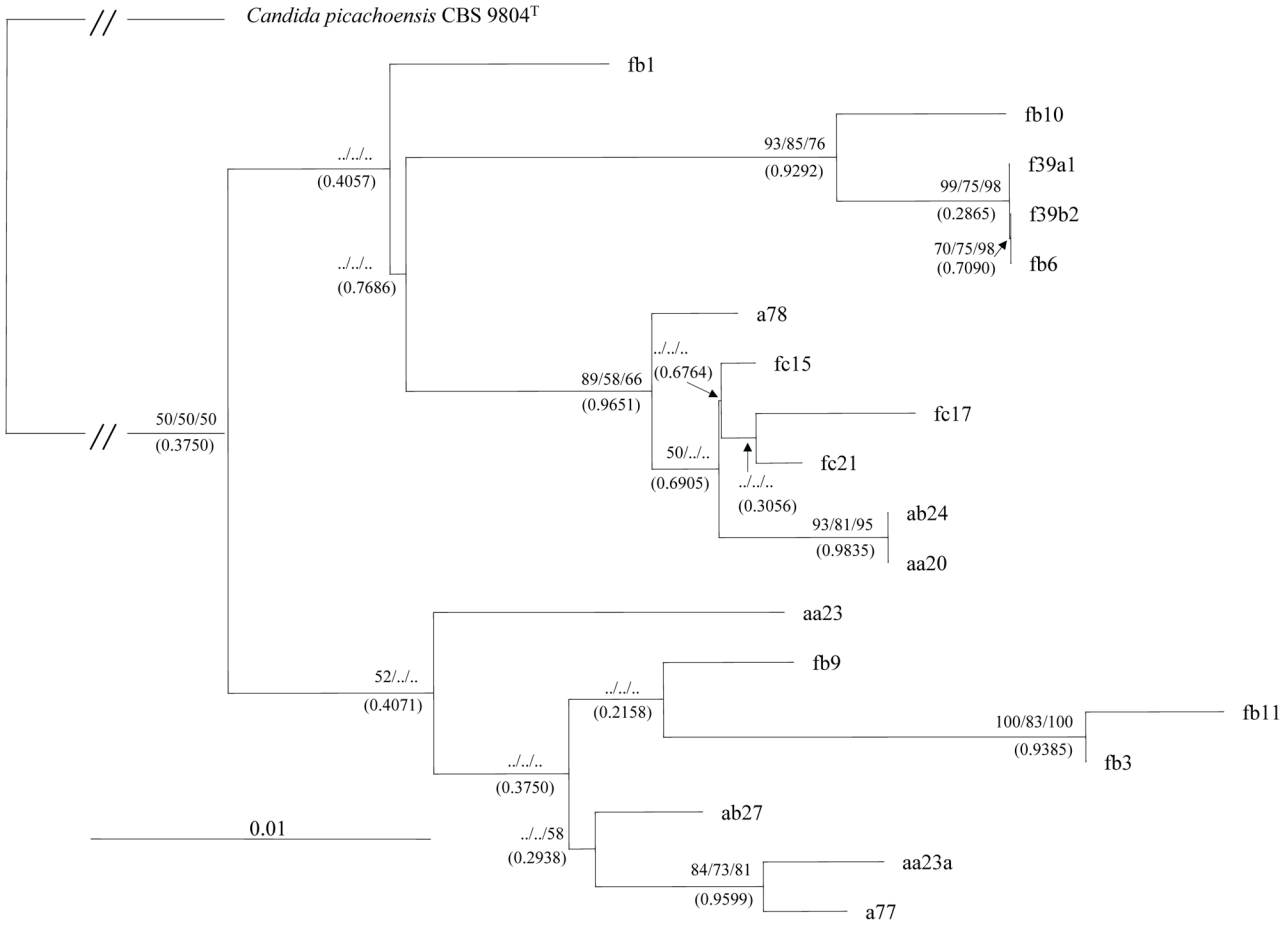
A phylogenetic tree derived from the neighbor-joining analysis of the cloned D1/D2 sequences. Neighbor-joining bootstrap values (before the first slash), maximum-parsimony bootstrap values (between slashes) and maximum-likelihood values (after the second slash) ≥50% are given at branch nodes. Numbers in brackets are Bayasian posterior probabilities. Outgroup: *Candida picachoensis*. GeneBank accession numbers of the sequences are listed in Table 1. Bar, 0.01 substitutions per nucleotide position.

In principle several models could be proposed by which an array of divers rDNA repeats could arise. One possibility is gradual spread of a “master repeat” by serial duplications during which the new copies acquire novel mutations in their inherited sequences. If this is the case, then the evolutionary history can be reconstructed by a phylogenetic analysis producing a bifurcating phylogenetic tree. Therefore we aligned the cloned sequences and analysed the alignments with neighbor-joining, maximum-parsimony and maximum likelihood methods. First we performed the analysis separately with the clones of the strains. The trees obtained had very low statistical supports. Then the two sets of sequences were pooled and analysed together. The topologies of the trees obtained were congruent but the statistical support of the majority of the branches was very low again (Fig. 4). The Bayesian analysis delivered very similar results: almost identical tree topology with nodes supported by very low posterior probabilities (Fig. 4). In Bayesian phylogenetics, confidence in evolutionary relationships is expressed as posterior probability - the probability that a tree or clade is true.

The sequences of the two species did not form separate branches although both trees consisted of two major clusters. Both clusters contained sequences from both species as if the two strains shared a common pool of LSU genes although in one of the branches a few *M. fructicola* sequences formed a separate sub-group. The low bootstrap and posterior probability values as well as the intermixing of *M. andauensis* and *M. fructicola* sequences suggested that the rDNA arrays of these strains did not evolve in a treelike way but rather in a reticulate way that cannot be represented by a bifurcating tree. It has been demonstrated by numerous studies that intraspecific and intragenomic evolutionary relationships are not hierarchical and the application of tree-constructing methods to their analysis can lead to poor resolution or inadequate representation of genealogical relationships (for a review see [35]).

### Visualisation of Reticulation in the Evolution of the Cloned D1/D2 Sequences

**Figure 5:**
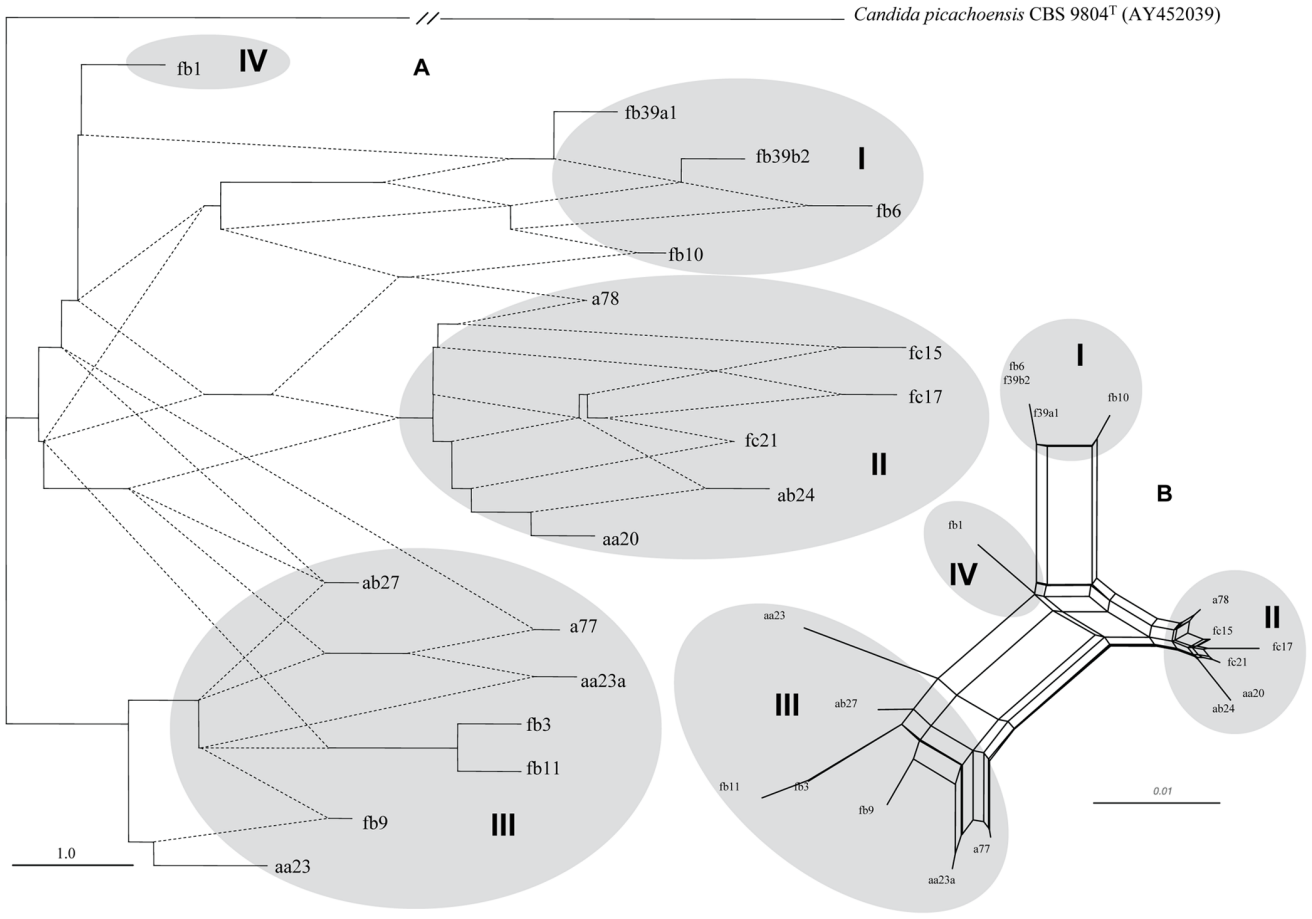
Network analysis of the cloned D1/D2 sequences. (A) Rooted rectangular phylogenetic network. Dotted lines represent network edges. (B) Neighbor-net splits graph. The scale bar represents the split support for the edges. Clusters described in the text are denoted by Roman numerals.

There exist several methods for verification and visualisation of reticulate events. We used SplitsTree4 V4.12.8 [14] to generate rooted rectangular phylogenetic networks (Fig. 5A). A phylogenetic network [36] is a rooted directed acyclic graph made up of so-called tree nodes, network nodes, tree edges and network edges. The tree nodes are nodes that are also seen in bifurcating phylogenetic trees (root, leaves and internal nodes at bifurcations). The network nodes (not seen in phylogenetic trees) are the nodes at which either edges converge or no bifurcation takes place. The tree edges are directed from tree nodes (root or internal nodes) towards tree nodes (internal nodes or leaves). The network edges are directed from tree nodes towards network nodes. In Fig. 5A the network edges are shown as dotted lines and the tree edges are continuous lines. The high number and the directions of network edges clearly demonstrate that reticulate events have played a major role in the evolution of the LSU rRNA genes of both strains. The same clones that formed the two large branches of the phylogenetic trees (Fig. 4) also grouped together in the phylogenetic network but their lineages were interconnected through network edges. With more careful inspection, four clusters could be recognised in the network which are designated I to IV in Fig. 5A. Three clusters had clones from both species which clearly demonstrates that the two type strains share a common LSU rRNA gene pool.

### Network Analysis

**Figure 6:**
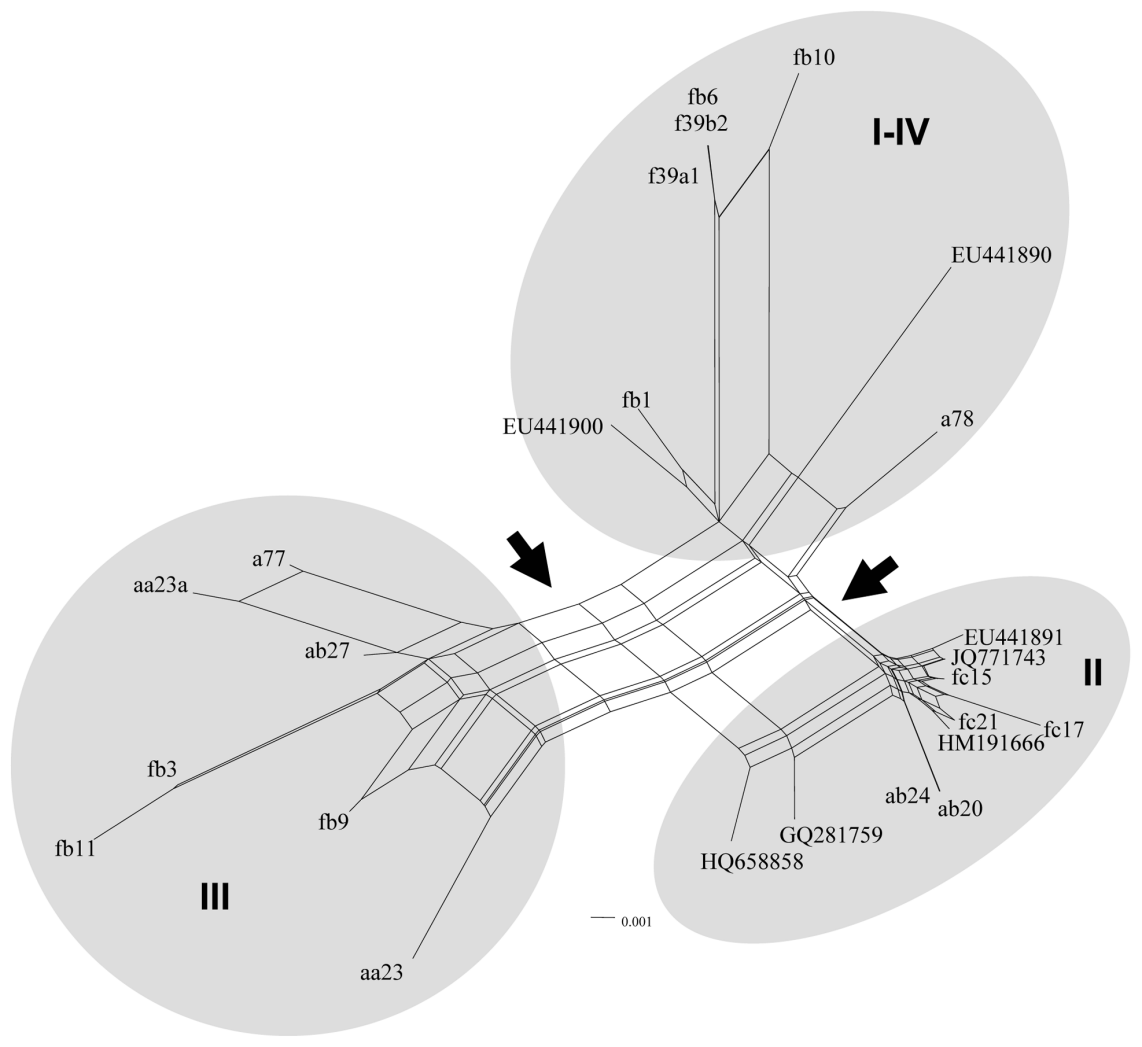
Neighbor-net splits graph of all cloned and database sequences. For display purposes bootstrap scores are not shown. The scale bar represents the split support for the edges. Arrows mark splits separating the three major clusters. Clusters described in the text are denoted by Roman numerals.

To explore the revealed interactions in greater detail, we subjected the multiple alignment of the cloned sequences to a neighbor-net network analysis. The neighbor-net method [14] based on the neighbor-joining algorithm [37] produces circular splits and uses a circular network algorithm [38] to get planar networks. A split is a partition of the set of data (sequences) into two groups. The set can be partitioned by numerous splits, and then a network can be built from these splits. Each split will define an edge connecting the two partitions. The outcome is a splits graph. Splits can be compatible and incompatible. Compatible splits correspond to branches in a phylogenetic tree, so the splits graph for a compatible collection of splits is a tree. An incompatible split separates nodes that are not connected with a branch in a tree. To generate a network, incompatible collections of splits must be allowed. Neighbor-net uses so-called “weakly compatible” splits [14]. When splits are incompatible (they define contradictory groupings), a box (cycle) is introduced to indicate that there are alternative splits. So, boxes in the splits graph can be used to locate reticulations. In a splits graph, a pair of nodes may be linked by a single edge (tree-like part) or a set of parallel edges depicting alternative evolutionary possibilities (reticulate part). The unrooted neighbor-net network we obtained (Fig. 5B) was distinctly non-treelike. The networked relationships among the sequences with box-like structures instead of bifurcations confirmed the notion that reticulation has occurred in their evolution. Even though the network was highly netted, distinct clusters could be discerned. Then we repeated the analysis also involving the database sequences deposited under the taxonomic name *M. fructicola* and obtained from strains different from the type strain (Table 1). None of these sequences had ambiguous sites. In contrast, the type strain and both *M. andauensis* database entries have such sites, so they were excluded from the analysis. The inclusion of the database sequences in the analysis slightly changed the overall topology of the network (Fig. 6) because it merged clusters I and IV and moved a78 from cluster II into the merged cluster. Two edges marked with arrows in Fig. 6 clearly separated the three clusters but not the two species because even the database *M. fructicola* sequences grouped into two clusters intermixed with sequences cloned from the *M. andauensis* type strain. Cluster II, containing both *M. andauensis* and *M. fructicola* clones had the highest number of conflicting splits. The presence of the boxes indicates reticulation but further analysis by other methods (e.g. PADRE: a package for analysing and displaying reticulate evolution [39], algorithms suitable for detection of recombination [30 and references therein], sequencing and structural analysis of the entire rDNA arrays) are needed to determine what processes are actually involved. We performed a Phi test [30] to detect recombination. This method examines “incompatibilities” in phylogenetic signals. If two lineages diverge and never recombine, then adjacent polymorphisms (or heterogeneity) will most likely be “compatible”, that is both polymorphic (or heterogeneous) sites will support the same tree topology (will have the same phylogenetic signal). If they do not support the same topology, they are termed „incompatible”. Incompatible sites can have two possible histories, one in which there was recurrent mutation in different lineages and one in which there was recombination between lineages. The Phi test found statistically significant evidence for recombination (*P* = 0.0021) in the alignment of the sequences.

## Discussion

The presence of ambiguous nucleotides in the database of D1/D2 sequences of the type strains of *M. andauensis* and *M. fructicola* suggested that either the collection cultures used for sequencing consisted of populations of cells of different genomes or the strains had diverse large subunit rRNA genes in their rDNA arrays. To find out which explanation was correct, we generated single-cell cultures from both strains and cloned D1/D2 domains from them. We assumed that if the sequence ambiguity was due to mixed cell populations in the collection cultures, then the single-cell cultures (yeast clones) should not have identical sequences and each of them should only have one type of D1/D2 sequence. We found that this was not the case because we could clone several different D1/D2 versions from the single-cell cultures. This result clearly demonstrated that the type strains of both species possessed heterogeneous rDNA arrays consisting of repeats of non-identical sequences.

So far, little attention has been devoted to intragenomic rDNA heterogeneity in yeasts because the ascomycetous yeasts are generally believed to have uniform rDNA repeat arrays due to homogenisation by concerted evolutions [40]. In fact this uniformity has become a sort of basic tenet of molecular taxonomy and is routinely exploited in taxonomic classification of strains, species delimitation and mapping of phylogenetic relationships (for a review see [6]). Exceptions are certain hybrid species (e.g. *Pichia sorbitophila*) and the alloploid and chimerical strains arisen from rare interspecies mating (e.g. in the genera *Saccharomyces* and *Zygosaccharomyces*) which have different versions of LSU rDNA repeat units inherited from homozygous strains of euploid parental species (e.g. [41], [42], [43], [44], [45]). Interspecific hybrids heterozygous for rDNA can also be produced under laboratory conditions but they are either sterile [46] or genetically unstable [47] which can unfavourably affect their chances to survive under natural conditions. In principle, the strains examined here could also be heterozygous hybrids because they are diploid as suggested by their ability to produce ascospores directly from cells (chlamydospores) without conjugation [3], [10]. However, the hybrids usually have only two parental alleles (e.g. [42]), whereas the *Metschnikowia* type strains examined here have at least 6 (*M. andauensis*) and 10 (*M. fructicola*) different large subunit rRNA alleles. We consider it more likely that they have mosaic arrays of rDNA repeats containing (most probably) paralogous D1/D2 segments differing by as many as 18 and 25 substitutions.

Intragenomic diversity of large subunit rDNA sequences have already been noticed in other ascomycetous yeasts. Lachance et al. [33] found mixtures of two D2 variants in certain *Clavispora lusitaniae* strains. In *Geotrichum candidum*, Alper et al. [48] identified ambiguous nucleotides in large subunit rDNA fragments amplified directly from 15 strains. However, in neither species was the phenomenon examined in greater detail. Since the genera *Clavispora* and *Metschnikowia* are members of the same family (Metschnikowiaceae) and phylogenetically related [49], the results obtained in this work can be considered an extension of the study of Lachance et. al [33]. Even the rDNA array of *S. cerevisiae* shows some intragenomic variability. Ganley and Kobayashi [50] found two polymorphic (heterogeneous) sites in the entire large-subunit rRNA genes. James et al. [51] detected altogether 35 (only 3 in D1/D2) polymorphic (heterogeneous) sites, but the level of variation differed by nearly an order of magnitude between individual strains. Neither study reported on cloning and comparison of individual repeats of the rDNA arrays.

Remarkably, both highly variable D1/D2 regions of the *Metschnikowia* type strains are located in segments that fold back to form hairpin loops in the predicted secondary structure of the LSU rRNA. The more variable VR2 region corresponds to a short stretch of an expansion segment of the *S. cerevisiae* D2 domain which is missing in the *E. coli* LSU rRNA molecule [32]. Similar expansions that increase the size of the domain at this position also occur in other fungi [52] including *C. lusitaniae* [33]. For example the D2 domain of the *Saccharomyces* large subunit rRNA is twice as large as the corresponding domain II of the *E. coli* large subunit rRNA [32]. The *Metschnikowia* D1/D2 domains studied here are only slightly shorter than that of *S. cerevisiae*. While the core regions of the LSU rRNAs are highly conserved structurally across all domains of life, the expansion segments evolve more rapidly [52], presumably due to reduced functional constraints. Consistently with this, the VR2 regions of the *Metschnikowia* sequences are more variable than their VR1 regions which reside in the core of the large subunit rRNA molecules.

A common feature of the predicted hairpins was that they contained almost all variable sites in the back-folding stretches. As the cloned *Metschnikowia* sequences differed in the number and location of the nucleotide substitutions in these regions, the secondary structures of their hairpins showed variable patterns of alternating helical and non-helical stretches. However, despite the internal variability, the size and the overall shape of the hairpins were alike in all clones. This structural stability can most probably be attributed to non-canonical base pairings and compensatory mutations taking place in the complementary stretches of the hairpin helices. We found that the non-canonical wobble base pair G:U was frequent in both variable regions. Since the thermodynamic stability of the wobble base pair G:U is comparable to that of the canonic G:C pair [34], the transitions between C and T in the coding DNA have less severe effect on the helical structure of the RNA stems than the A-G transitions or the transversions that either disrupt base pairings or create new pairs. This difference can explain why the majority of the variable sites alternate between C and T. The other factor that can stabilise the stem structure is the occurrence of compensatory mutations in sites that pair with the variable nucleotides of the back-folding stretch. We found three pairs of variable sites in which substitutions can take place in a concerted way to maintain base pairing. It is tempting to speculate that because of the little effect on rRNA structure, the alternative versions of variable sites of VR1 and VR2 are not selected out by the evolution and can persist simultaneously in the same genome. Nucleotides in other parts of the rRNA molecules may not enjoy a similar protection.

How can the cell cope with the presence of a mixed population of different 26S rRNA molecules without severe consequences in fitness? The most obvious answer is that due to the attenuating effect of wobble pairing and compensatory mutations, the RNA molecules encoded by the different genes of the array have quite similar secondary structures and may not significantly differ in functional activity either. However, there might also be other factors to be taken in consideration. A number of studies have shown that not all repeats of the rDNA array are used in the cell. For example, in *S. cerevisiae* only half of the repeats are actively transcribed [53], [54]. It was hypothesized that those that are not transcribed reside in regions silenced by the chromatin structure [55]. If this is the case in the *Metschnikowia* strains as well, then certain large subunit rDNA gene variants may remain silent and the RNA molecules produced may consequently be less diverse than the genes of the rDNA array.

The question arises as to whether the persistence of different large subunit rRNA genes within one genome is a peculiarity of a few yeast strains or can be a more wide-spread, albeit overlooked phenomenon. To address this question one should test a large number of other yeasts species for D1/D2 homogeneity. Here we only examined the database sequences deposited by other authors as D1/D2 domains of *M. andauensis* or *M. fructicola*. The sequence of the only non-type *M. andauensis* strain had even more ambiguous sites than the type strain. In contrast, the 6 *M. fructicola* sequences were free of ambiguous nucleotides. This difference might be interpreted as indicating that the latter species has both heterogeneous and non-heterogeneous strains. However, even a heterogeneous strain may produce an “error-free” sequence if one of the repeat versions predominates over the other versions.

The extent of the intragenomic divergence of the D1/D2 domains further suggests that the *Metschnikowia* type strains fail to operate the mechanism of concerted evolution of rDNA which functions efficiently in the vast majority of organisms. Concerted evolution of the rDNA repeats ensures that a mutation that arises in one copy is eliminated or spreads by inter-copy interactions through the array until fixation [56]. For example, in *S. cerevisae* the rDNA repeats undergo rapid homogenization: the new mutations are either deleted or multiplied during continual unequal recombination until one variant finally becomes dominant [50]. The large number of different D1/D2 sequences in the genomes of the *Metschnikowia* strains indicates that in these organisms concerted evolution must be very inefficient and the ribosomal genes may not evolve in a strictly concerted manner. Few other organisms are known in which concerted evolution of rDNA seems to operate with low efficiency. Besides the two yeasts mentioned above, the grasshopper *Podisma pedestris*, the flatworm family Dugesiidae and certain aphid species were found to have heterogeneity in their rDNA arrays (reviewed in [57]). It was proposed that the rDNA units of these organisms simply escaped the process of concerted evolution or were under selective pressure to evolve variable rDNA genes.

The intragenomic D1/D2 sequence diversity of the *Metschnikowia* type strains exceeds considerably the intraspecies diversity observed by Kurtzman and Robnett [31] in ascomycetous yeasts. These authors found that conspecific yeast strains rarely differed by more than three substitutions and a difference higher than 1% between two strains indicated that the strains might have belonged to different species. On that basis one could conclude that the rDNA arrays of the two *Metschnikowia* type strains are polyphyletic and consist of genes brought together by interspecies hybridisation events. However, other authors revealed much larger differences between conspecific strains and in heterozygous or heterogeneous strains. For example the D2 sequences of *C. lusitaniae* strains were found to differ by as many as 32 substitutions [33], more than the highest number of substitutions (25) detected in this study between the least similar *Metschnikowia* clones. In view of these observations, it seems to be more plausible that reticulate evolution involving intraspecies hybridisation and intragenomic events such as recombination, gene conversion, deletion, duplication, etc. might have shaped the rDNA arrays of both strains.

Although the intragenomic processes leading to homogenisation seem to be rather inefficient in the *Metschnikowia* type strains, reticulation does take place in their genomes as demonstrated by the poor statistical support of the neighbour-joining and maximum-parsimony phylogenetic trees and the topologies of the phylogenetic networks and the neighbour-net splits graphs. In addition, when the cloned sequences of the two strains were pooled before the analyses, the phylogenetic trees and the networks did not group them into separate clusters. Presumably, the strains could have exchanged parts of their rDNA arrays by hybridisation and recombination and their rRNA arrays did not evolve separately but in interaction. The pair-wise homoplasy (Phi) test confirmed that recombination did play a role in their evolution. The presence of D1/D2 domains of identical sequences in the type strains also hints towards interspecies hybridisation events. Of course, this conclusion can only be correct under the premise that the autogamy proposed for the *M. pulcherrima* subclade [7] does not pose an impenetrable barrier to sexual interactions. Addition of database *M. fructicola* sequences to the analysed set of sequences did not change significantly the topology of the neighbour-net splits graphs: the *M. andauensis* and *M. fructicola* sequences remained intermixed and did not form separate clusters. Thus, even the repeats of the non-type strains fit into the hypothesized common pool of rDNA repeats. The lack of a sharp discontinuity between their sequences implies that *M. andauensis* and *M. fructicola* are not reproductively isolated and their distinct taxonomic status should be revised. It has to be noted here that Lachance et al. [58] already expressed doubts regarding the taxonomic division of the *M. pulcherrima* clade to which these species belong because unambiguous assignment of its strains to species on the basis of their D1/D2 was not always possible (e.g. [3], [11]) and the species of the clade were virtually indistinguishable from each other by physiological tests [8]. If the phenomenon described in this study also characterizes other yeast species, it should be taken into consideration in the taxonomic division of yeasts, their barcoding and in biodiversity research using barcodes such as metagenomics.
